# 3,4′,5-Trimethoxy-*trans*-stilbene Alleviates Endothelial Dysfunction in Diabetic and Obese Mice via Activation of the AMPK/SIRT1/eNOS Pathway

**DOI:** 10.3390/antiox11071286

**Published:** 2022-06-28

**Authors:** Chunxiu Zhou, Yi Tan, Baojun Xu, Yitao Wang, Wai-San Cheang

**Affiliations:** 1 State Key Laboratory of Quality Research in Chinese Medicine, Institute of Chinese Medical Sciences, University of Macau, Avenida da Universidade, Taipa, Macau 999078, China; mc05835@um.edu.mo (C.Z.); mc05845@um.edu.mo (Y.T.); ytwang@um.edu.mo (Y.W.); 2 Food Science and Technology Program, BNU-HKBU United International College, Zhuhai 519087, China; baojunxu@uic.edu.cn

**Keywords:** 3,4′,5-trimethoxy-*trans*-stilbene, endothelial dysfunction, diabetes, endoplasmic reticulum stress, oxidative stress

## Abstract

3,4′,5-trimethoxy-*trans*-stilbene (TMS) is a methoxylated derivative of resveratrol. Previous studies showed the vaso-protective effects of resveratrol; nevertheless, research on this derivative is scarce. The current study aimed to explore whether TMS can alleviate endothelial dysfunction in diabetic and obese mice, along with the underlying mechanisms. Thoracic aortas isolated from male C57BL/6J mice and primary cultures of rat aortic endothelial cells were treated with high glucose with or without TMS. High glucose exposure impaired acetylcholine-induced endothelium-dependent relaxations, down-regulated NO bioavailability and the AMP-activated protein kinase (AMPK)/Sirtuin 1 (SIRT1)/endothelial nitric oxide synthase (eNOS) pathway, increased endoplasmic reticulum (ER) stress and oxidative stress, which were reversed by TMS treatment. Moreover, the protective effects of TMS were abolished by Compound C (AMPK inhibitor), and EX527 (SIRT1 inhibitor). The mice were fed with high-fat diet (60% kcal% fat) for 14 weeks to establish a diabetic and obese model, and were orally administered TMS (10 mg/kg/day) in the last 4 weeks. Chronic TMS treatment alleviated endothelial dysfunction via enhancing the AMPK/SIRT1/eNOS pathway and attenuated oxidative stress and ER stress in aortas of diet-induced obese mice. In summary, our study reveals the potent vaso-protective effect of TMS and its therapeutic potential against endothelial dysfunction in metabolic disorders.

## 1. Introduction

Diabetes is a metabolic disease mainly characterized by hyperglycemia [1]. Apart from hypertension, hyperglycemia is the second most common risk factor, and can result in endothelial dysfunction, impaired vasodilation, oxidative stress, inflammation, and abnormality in vascular smoooth muscle cells (VSMCs) [2,3]. Vascular endothelial cells (ECs) are the main targets of hyperglycemic damage as they cannot modulate intracellular glucose concentration with respect to blood glucose levels [4]. Until now, extensive evidence from previous studies reveals the close correlation between inflammatory risks and cardiovascular diseases including endothelial dysfunction in diabetes [5]. Both oxidative stress and endoplasmic reticulum (ER) stress can trigger inflammation in ECs and thereby contribute to endothelial dysfunction.

**Figure 1:**
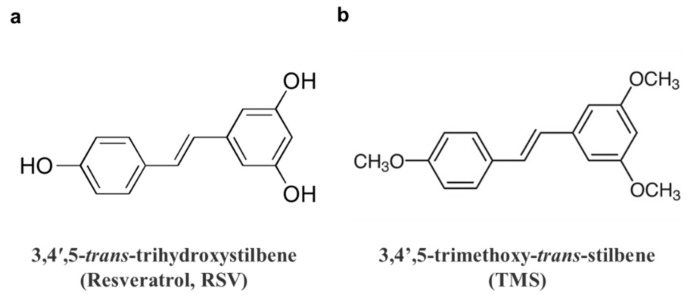
Chemical structures of (**a**) 3,4′,5-*trans*-trihydroxystilbene (resveratrol, RSV), (**b**) 3,4′,5-trimethoxy-*trans*-stilbene (TMS).

Resveratrol (3,4′,5-*trans*-trihydroxystilbene, RSV; Figure 1a is a natural non-flavonoid polyphenol present in many plant-based foods and beverages, including peanuts, cranberries, blueberries and grapes. Numerous reports have described its therapeutic and health-promoting benefits. Among all these effects, research on the anti-inflammatory and cardiovascular effects of RSV is always a hot topic. Based on its strong ability to reduce inflammation, it is also increasingly regarded as an anti-aging therapeutic in many aging diseases, especially metabolic disorders like type 2 diabetes [6,7]. RSV exerts many cardiovascular benefits including stress resistance, energy metabolism, circadian clock, microbiota composition, exercise mimetic, and longevity regulation [8]. Despite these remarkable health benefits, some pharmacokinetic drawbacks limit its applications in effective treatments, including fast transformation, low bioavailability, and instability. As a result, many researchers have paid attention to its structural modification. Compared to the three hydroxyl groups of RSV, its methoxylated derivatives exhibit increased lipophilic properties, higher binding affinity with membrane proteins, higher availability, and higher stability [9]. In recent years, natural or synthetic RSV derivatives have been studied, especially the methoxylated ones that have exerted diverse therapeutic potentials. A natural methoxylated derivative of RSV is 3,4′,5-trimethoxy-*trans*-stilbene (TMS; Figure 1b). Previous studies have revealed some potent therapeutic effects of TMS against inflammation in macrophages, invasiveness in breast cancer cells, high glucose-induced endothelial dysfunction, and apoptosis [10,11,12,13,14]. However, the investigation of the beneficial effects of TMS against endothelial dysfunction, particularly the underlying mechanism is incomplete and remains to be explored.

Sirtuin 1 (SIRT1) is a nicotinamide adenine dinucleotide (NAD^+^)-dependent deacetylase and regulates many genes involved in metabolic processes, including fatty acid oxidation, mitochondrial function, and gluconeogenesis. In ECs, SIRT1 can deacetylate endothelilal nitric oxide synthase (eNOS) to increase the enzymatic activity of eNOS and thereby enhance nitric oxide (NO) production and endothelium-dependent relaxation (EDRs) [15]. AMP-activated protein kinase (AMPK) is proven to upregulate SIRT1 activity and the deacetylation mediated by SIRT1, through increasing intracellular NAD^+^ [16,17]. AMPK activation and SIRT1 activation have been demonstrated to be protective in the cardiovascular system. For instance, AMPK activation induced by metformin treatment [18] or by endurance exerise [19] alleviates ER stress and oxidative stress in ECs to combat diabetes-associated vascular dysfunction; and resveratrol-induced SIRT1 activation also improves vascular function in diabetes [20]. ER stress is linked with cardiovascular diseases; and in ECs, many risk factors including hyperglycemia, oxidized phospholipids, homocysteine, and hexosamine all contribute to ER stress, which has been proven by the activation of three ER-resident transmembrane proteins, such as activating transcription factor 6 (ATF6), protein kinase RNA-like ER kinase (PERK), and inositol-requiring enzyme 1 (IRE1) [21,22]. ER stress in ECs leads to decreased phosphorylation of eNOS and thus NO production, as well as elevated oxidative stress, resulting in endothelial dysfunction.

In the present study, we aimed to investigate the vaso-protective effect of TMS in attenuating endothelial dysfunction and ER stress associated with diabetes both in vitro and in vivo; and to identify the underlying molecular mechanims.

## 2. Materials and Methods

### 2.1. Materials

TMS (purity > 98%; Cat#T1829) and RSV (purity > 98%; Cat#0071) were purchased from Tokyo Chemical Industry Co., Ltd. (Tokyo, Japan) Low-glucose Dulbecco’s modified Eagle’s medium (DMEM) powder (Cat#31600034), Roswell Park Memorial Institute (RPMI) 1640 medium powder (Cat#31800022), fetal bovine serum (FBS; Cat#10270106), penicillin-streptomycin (10000 U/mL; Cat#15140122), and 0.5% trypsin-EDTA (10×; Cat#15400054) were obtained from Gibco (Carlsbad, CA, USA). Heparin sodium salt (Cat#A16198) was purchased from Alfa Aesar (Carlsbad, CA, USA). Thiazolyl blue tetrazolium bromide (MTT; Cat#M6494) and bovine serum album (BSA; Cat#A8022) were obtained from Sigma Aldrich (St Louis, MO, USA). Dihydroethidium (DHE; Cat#D11347) was purchased from Invitrogen (Carlsbad, CA, USA).

### 2.2. Isolation and Primary Culture of Rat Aortic Endothelial Cells (RAECs)

RAECs were isolated from thoracic aortas of Sprague-Dawley (SD) rats through an enzymatic digestion method. Rats were euthanized by CO_2_ suffocation. Aortas were then immediately removed and dissected in sterile PBS. Incubating and digesting the aortas in sterile PBS containing collagenase type 1A (2 mg/mL, Sigma Aldrich; Cat#C9891) solution at 37 °C for 15 min with gentle shaking detached the endothelial cells, which were then collected by centrifugation at 2500 rpm for 10 min and re-suspended in RPMI-1640 containing 10% FBS, plus 100 U/mL penicillin and 100 µg/mL streptomycin. After incubation at 37 °C for 1 h, the medium was refreshed. RAECs were then incubated until the cells reached 80–90% confluency in a humidified atmosphere containing 5% CO_2_ at 37 °C.

### 2.3. Ex Vivo Culture of Mouse Aortas

After the mice were euthanized by CO_2_ suffocation, the thoracic aortas were isolated rapidly and cleaned from surrounding fat and adjacent connective tissues in sterile PBS. Arteries were then cut into ring segments and were incubated in DMEM supplemented with 10% FBS plus 100 U/mL penicillin and 100 µg/mL streptomycin at 37 °C in a humidified incubator with 5% CO_2_. The segments were incubated in the normal medium with mannitol as osmotic control, high glucose (30 mM) or high glucose plus various drugs including different concentrations of TMS, RSV, Compound C (Cpd C, 5 μM, AMPK inhibitor, Sigma-Aldrich; Cat#171260), and selisistat (EX527, 1 μM, SIRT1 inhibitor, Beyotime; Cat# SC0281) in an incubator at 37 °C for 48 h. The ring segments were then collected for further functional studies using a wire myograph, and Western blotting assays. The culture medium of the aortic segments was also collected for testing NO release.

### 2.4. Animals and Drug Treatment

The use of animals and related research protocols were approved by the Animal Research Ethical Committee, University of Macau, and was consistent with the Guide for the Care and Use of Laboratory Animals. Male C57BL/6J mice and SD rats were purchased from the faculty of Health Science Animal Centre of University of Macau and housed in animal holding rooms with controlled temperature (22–23 °C) plus an alternating 12 h light/dark cycle. The mice were fed with a high-fat diet (60% kcal% fat) for 10 weeks starting at the age of 6 weeks to establish a type 2 diabetic and obese model followed by oral administration of vehicle (Kolliphor HS-15, Sigma-Aldrich; Cat#42966), and TMS at 10 mg/kg/day for another 4 weeks. Mice fed with a normal chow diet acted as control.

### 2.5. Blood Pressure Measurement

After resting for 10 min, the systolic (SBP) and diastolic (DBP) blood pressure of the mice were measured using the CODA noninvasive blood pressure system (Kent Scientific Corporation, Torrington, CT, USA) in a tail-cuff method.

### 2.6. Blood Glucose Measurement

After fasting for 6 h, fasting blood glucose (FBG) level was tested by the blood drawn from the mouse tail using a commercial glucometer.

### 2.7. Isometric Force Measurement in Wire Myograph

The aortic segments (~2 mm) were suspended in a Multi Myograph System (Danish Myo Technology, Denmark) for recording of the changes in isometric tension. The aortic rings were fixed by 2 steel wires (40 μm in diameter) which were inserted through the lumen of the vessel, and then fixed to the jaws of the myograph chamber. Each organ chamber was gassed by 95% O_2_ plus 5% CO_2_ and filled with 5 mL Krebs solution (mM): 119 NaCl, 4.7 KCl, 2.5 CaCl_2_, 1 MgCl_2_, 25 NaHCO_3_, 1.2 KH_2_PO_4_, and 11 glucose (pH 7.4). The rings were stretched to 3 mN, an optimal baseline tension and then stabilized for 60 min before being contracted by 60 mM KCl. The first series of the experiments studied the alteration in endothelium-dependent relaxations (EDRs). Rings were contracted by phenylephrine (Phe, 3 μM, Sigma-Aldrich; Cat#P6216) to establish a stable tension and then relaxed by acetylcholine (ACh, 3 nM–10 μM, Sigma-Aldrich; Cat#A6625) cumulatively. The second set examined the endothelium-independent relaxations. Aortic rings were first treated with 100 μM N^G^-nitro-L-arginine methyl ester (L-NAME, a NOS inhibitor, Sigma-Aldrich; Cat#483125) for 30 min to eliminate the interference of endothelium-derived NO, and then contracted by 3 μM Phe. The relaxations in response to sodium nitroprusside (SNP, an exogenous NO donor, 1 nM–10 μM, Sigma-Aldrich; Cat#567538) were then measured.

### 2.8. Cell Viability Analysis

The effects of TMS and RSV on the cell viability were determined by MTT assay. RAECs were seeded into 96-well plates (7 × 10^3^ cells/well, 100 μL medium/well). After incubating overnight, the cells were treated with TMS and RSV at final concentrations of 1, 10, 20 and 50 μM for another 48 h. The TMS and RSV were dissolved in RPMI 1640 medium. Thereafter, the MTT reaction solution (20 μL, 5 mg/mL) was added into each well, followed by 3 h of incubation at 37 °C. Finally, the supernatants were removed and the formazan crystals in each well were dissolved in 150 μL of DMSO. After being shaken for 30 min, the 96-well plates were read by a SpectraMax M5 microplate reader (Molecular Devices, Silicon Valley, CA, USA) at 570 nm to measure absorbance.

### 2.9. Detection of NO Production in Cultured Medium

The NO levels of collected medium from cultured RAECs and mouse aortas were measured by the Griess reagent kit (Invitrogen; Cat#G7921) according to the manufacturer’s instructions. The absorbance was detected at 548 nm using the microplate reader.

### 2.10. Detection of ROS by Dihydroethidium (DHE) Staining

Ring segments of mouse aortas were frozen in OCT compound (Tissue-Tek, Sakura Finetek; Cat#16374) and sliced into sections of 10-μm thickness by a Leica CM 1000 cryostat. The arterial sections and treated RAECs were incubated in DHE (5 μM)-containing NPSS in dark at 37 °C for 30 min. The fluorescence level of DHE was determined by the fluorescence shift using a Leica-DMi8 inverted fluorescent microscope. Densitometry analysis was performed by the Image-Pro Plus 6.0 software.

### 2.11. Western Blotting Analysis

Aortas were snap frozen by liquid nitrogen and then homogenized in ice-cold RIPA lysis buffer (Beyotime; Cat#P0013C) plus cOmplete protease inhibitor cocktail (Roche; Cat#04906845001) and PhosSTOP phosphatase inhibitors (Roche; Cat#04693116001) and the lysates were incubated on ice for 30 min. After treatment, RAECs were harvested on ice and lysed with RIPA solution containing 1% Phosphatase Inhibitor Cocktail (Thermo; Cat#78427) and 1% phenylmethylsulfonyl (PMSF, Thermo; Cat#36978). The lysates were centrifuged at 15,000 rpm for 20 min at 4 °C to collect supernatants. The total protein content was determined by a BCA protein assay kit (Beyotime; Cat#P0011). Protein samples were separated by 8% or 10% SDS-PAGE gels and the blots were transferred to PVDF membranes (Millipore, Billerica, MA, USA; Cat#IPVH00010). The membrane was blocked by 1% BSA in TBST buffer and then incubated with appropriate primary antibodies overnight at 4 °C and with secondary antibodies for another 2 h at room temperature. Finally, the specific protein bands were visualized with a Supersignal^TM^ West Femto Maximum Sensitivity Substrate (Thermo Scientific, Allentown, PA, USA; Cat#34095) and scanned by a ChemiDoc^TM^ MP Imaging System (BIO-RAD, Hercules, CA, USA). The following primary antibodies were used: phospho-eNOS (Ser1177) rabbit mAb (1:500, Cell Signaling Technology; Cat#9570), eNOS rabbit mAb (1:1000, Cell Signaling Technology; Cat#32027), phospho-AMPKα (Thr172) rabbit mAb (1:1000, Cell Signaling Technology; Cat#50081), AMPKα antibody (1:500, Cell Signaling Technology; Cat#2532), phospho-eIF2α (Ser51) rabbit mAb (1:1000, Cell Signaling Technology; Cat#3398), eIF2α rabbit mAb (1:500, Cell Signaling Technology; Cat#5324), SirT1 mouse mAb (1:500, Cell Signaling Technology; Cat#8469), GAPDH rabbit mAb (1:3000, Cell Signaling Technology; Cat#5174), anti-ATF6 antibody (1:1000, abcam; Cat#ab203119), recombinant anti-XBP1 antibody (1:1000, abcam; Cat#ab220783), anti-ATF3 Antibody (1:500, Santa Cruz; Cat#sc-518032), and anti-GRP78 antibody (1:500, Santa Cruz; Cat#sc-166490).

### 2.12. Statistical Analysis

All data of experiments are showed as mean ± standard error of mean (SEM) of n independent experiments. Variance among multiple groups was analyzed using one-way analysis of variance (ANOVA) followed by an unpaired Student t test using GraphPad Prism software (GraphPad Software, San Diego, CA, USA). *P* < 0.05 is considered to be statistically significant.

## 3. Results

### 3.1. TMS Ameliorates Endothelial Dysfunction in Hyperglycemic Conditions

**Figure 2:**
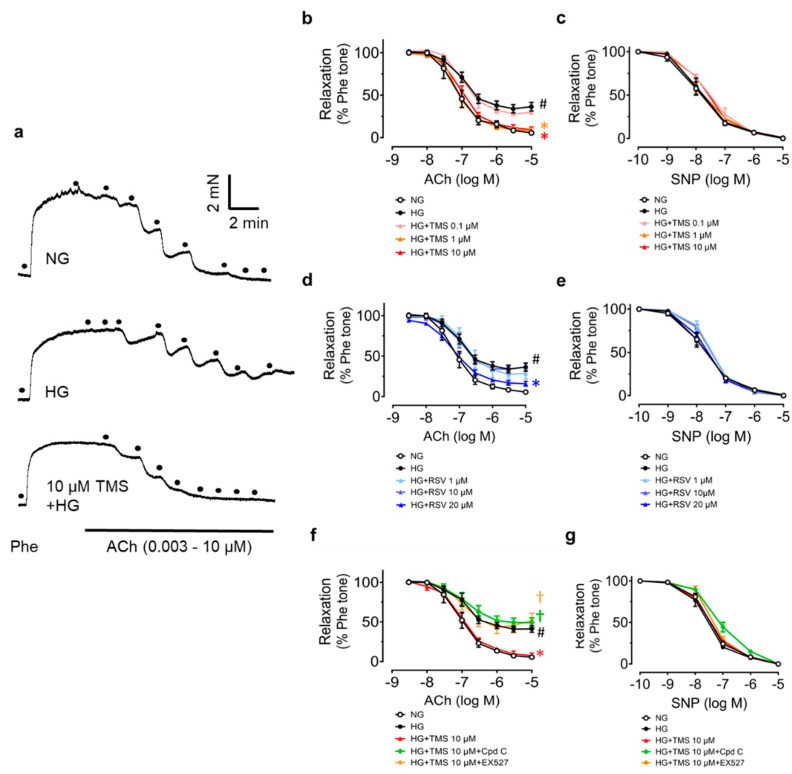
Protective effect of TMS and RSV on endothelium-dependent vasodilation in aortas from C57BL/J mice in a hyperglycemic condition. (**a**) Representative traces and (**b**,**d**) summarized data showing that high glucose (HG, 30 mM, 48 h) impaired acetylcholine(ACh)-induced endothelium-dependent relaxation as compared to normal glucose (NG, 5.55 mM with mannitol), and that different concentrations of TMS and RSV improved the relaxation to varying extents. Effects of AMPK inhibitor Compound C (Cpd C, 5 μM) and SIRT1 inhibitor selisistat (EX527, 1 μM) on (**f**) ACh-induced relaxations in mouse aortas. (**c**,**e**,**g**) Sodium nitroprusside (SNP)-induced endothelium-independent relaxation in aortas. Data are expressed as the mean ± SEM, n = 4. # *P* < 0.05 vs. NG; * *P* < 0.05 vs. HG; † *P* < 0.05 vs. HG + TMS 10 μM.

The vaso-protective effect of RSV has been proven previously, so we wondered whether TMS had the same vaso-protective effect as RSV. Studies on ex vivo cultures of mouse aortas were performed first. Mouse aortas from C57BL/6J mice were incubated with high glucose (30 mM, 48 h) to establish a similar hyperglycemic condition to diabetes. Compared to the normal glucose (5.55 mM with mannitol as osmotic control in DMEM), the ACh-induced EDRs were impaired in high glucose-treated aortas. Treatment with TMS and RSV reversed the impairment in a dose-dependent manner (Figure 2a,b,d). Apparently, TMS exhibited a more potent vaso-protective effect than RSV. RSV had no effect at 1 and 10 μM and was effective at 20 μM while TMS remarkably, repaired the damage at 1 μM and 10 μM. SNP-induced endothelium-independent relaxation was unaltered among groups, indicating the normal response of vascular smooth muscle to NO (Figure 2c,e). Consequently, 10 μM of TMS was chosen as the effective concentration for further studies.

According to the previous studies on RSV, AMPK inhibitor Compound C (Cpd C, 5 μM) and SIRT1 inhibitor selisistat (EX527, 1 μM) were employed in co-incubation with TMS to explore its underlying mechanisms. The protective effect of TMS against high glucose-induced impairment of EDRs was abolished both by Compound C and EX527 (Figure 2f). Meanwhile, SNP-induced endothelium-independent relaxation remained unaltered (Figure 2g). It indicated that both AMPK and SIRT1 mediated the vaso-protective effect of TMS.

### 3.2. TMS Stimulates the AMPK/SIRT1/eNOS Pathway and Reduces ER Stress in Mouse Aortas Exposed to Hyperglycemic Conditions

**Figure 3:**
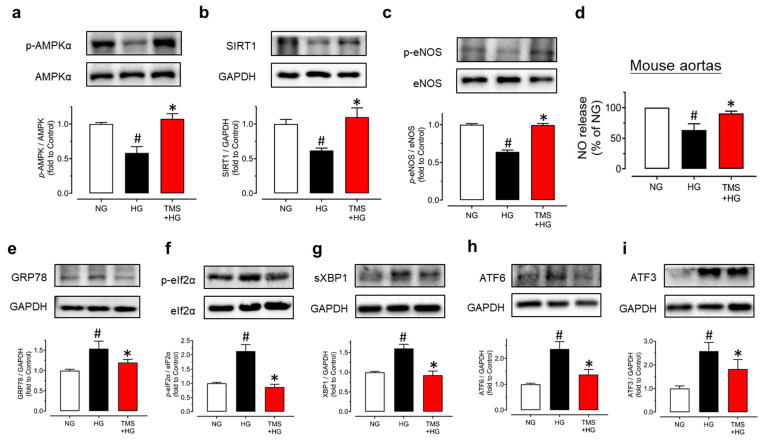
TMS activates the AMPK/SIRT1/eNOS pathway and reduces ER stress in mouse aortas in hyperglycemic conditions. Representative blots and summarized data showing (**a**) phosphorylation of AMPKα at Thr172 (p-AMPKα; 62kDa), (**b**) expression of SIRT1 (120 kDa) and (**c**) phosphorylation of eNOS at Ser1177 (p-eNOS; 140 kDa) as compared to their corresponding total protein or GAPDH (37 kDa) in mouse aortas treated with high glucose (HG, 30 mM) and TMS (10 μM) for 48 h. (**d**) NO release from aortas upon high glucose stimulation and co-treatment with TMS (10 μM) as determined by measuring the nitrite level in culture medium. Representative blots and summarized data showing ER stress markers including (**e**) GRP78 (78 kDa), (**f**) phosphorylation of eIF2α at Ser52 (p-eIF2α; 38 kDa), (**g**) spliced XBP1 (sXBP1; 56 kDa), (**h**) cleaved ATF6 (50 kDa) and (**i**) ATF3 (21 kDa) compared to its total protein or GAPDH in mouse aortas. Data are expressed as the mean ± SEM, n = 4. # *P* < 0.05 vs. NG; * *P* < 0.05 vs. HG.

For further verification of the underlying mechanisms, we isolated mouse aortas and treated them with high glucose (30 mM) and TMS (10 μM) for 48 h. Exposure to high glucose resulted in the significant reduction in the phosphorylation of AMPKα at Thr172, the phosphorylation of eNOS at Ser1177, and expression of SIRT1 in mouse aortas (Figure 3a–c). Notably, co-incubation with TMS normalized these changes. As the AMPK/SIRT1/eNOS pathway promotes NO bioavailability, the nitrite level in the conditioned culture medium of aortas was measured to confirm the effect. We found that the nitrite level was decreased by high glucose stimulation and was upregulated significantly by TMS treatment (Figure 3d). Taken together, TMS treatment might activate the AMPK/SIRT1/eNOS pathway to enhance NO bioavailability and protect against high glucose-induced endothelial dysfunction.

Importantly, high glucose stimulation also contributed to ER stress in the aortas as shown in our results (Figure 3e–i). TMS treatment suppressed the high glucose-induced ER stress markers including ER chaperone glucose-regulated protein 78 (GRP78), phosphorylation of eukaryotic initiation factor-2α (eIF2α) at Ser52, spliced X-box binding protein 1 (sXBP1), cleaved ATF6 and ATF3 in mouse aortas. All the findings suggested the effect of TMS against high glucose-induced ER stress was is in parallel with the vaso-protective effect.

### 3.3. TMS and RSV Alleviate High Glucose-Induced Oxidative Stress in Raecs without Affecting Cell Viability

**Figure 4:**
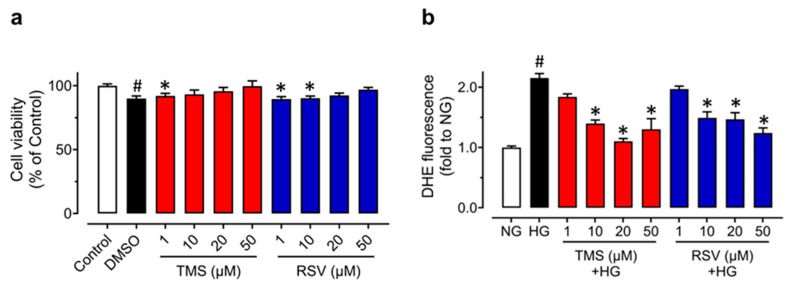
Effects of TMS and RSV on the cell viability and high glucose-induced oxidative stress in RAECs. (**a**) Cell viability of TMS and RSV in RAECs was assessed by the MTT assay. The cells were treated with different concentrations (1, 10, 20, 50 μM) of TMS and RSV for 48 h. (**b**) Inhibitory effects of TMS and RSV (1, 10, 20, 50 μM) on high glucose-induced (44 mM, 48 h) oxidative stress in RAECs as compared to the control (normal glucose, NG; 11.11 mM glucose in RPMI 1640 plus mannitol as osmotic control). Data are expressed as the mean ± SEM, n = 3. # *P* < 0.05 vs. NG. * *P* < 0.05 vs. HG.

In regard to the protective effect of TMS against endothelial dysfunction and ER stress induced by high glucose in mouse aortas ex vivo, we cultured primary RAECs to further confirm the effects in endothelial cells. The MTT assay was first performed to ensure there were no effects of TMS and RSV on cell viability. RAECs were treated with TMS and RSV at different concentrations (0, 1, 10, 20 and 50 μM) for 48 h. The results showed that TMS and RSV had a very minor effect on the cell viability of RAECs (Figure 4a). Therefore, we further explored the effects of TMS on RAECs treated with high glucose (44 mM, 48 h) at the safe concentrations of 10 and 50 μM.

To compare the anti-oxidative effects of TMS and RSV in RAECs, DHE fluorescence was measured (Figure 4b). High glucose (44 mM, 48h) stimulation increased ROS generation, which was reversed by TMS and RSV at concentrations of 10, 20, 50 μM, while there was no significant effect at 1 μM. These results indicated that TMS and RSV have a similar potency to suppress oxidative stress induced by high glucose.

### 3.4. TMS Activates the AMPK/SIRT1/eNOS Pathway and Alleviates ER Stress in Raecs Treated with High Glucose

**Figure 5:**
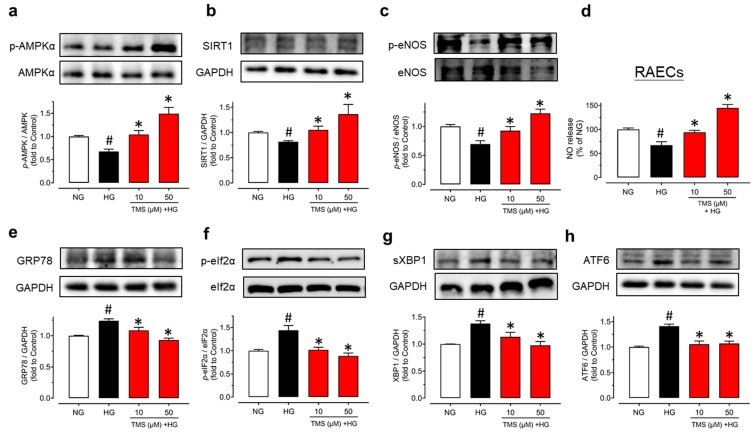
TMS stimulates the AMPK/SIRT1/eNOS pathway and attenuates ER stress in RAECs. Representative blots and summarized data showing (**a**) phosphorylation of AMPKα at Thr172 (p-AMPKα; 62 kDa), (**b**) expression of SIRT1 (120 kDa) and (**c**) phosphorylation of eNOS at Ser1177 (p-eNOS; 140 kDa) as compared to their corresponding total protein or GAPDH (37 kDa) in RAECs treated with high glucose (HG, 44 mM) and TMS (10 and 50 μM) for 48 h. (**d**) NO release from RAECs upon high glucose stimulation and co-treatment with TMS (10 and 50 μM) as determined by measuring the nitrite level in culture medium. Representative blots and summarized data showing ER stress markers including (**e**) GRP78 (78 kDa), (**f**) phosphorylation of eIF2α at Ser52 (p-eIF2α; 38 kDa), (**g**) spliced XBP1 (sXBP1; 56 kDa), and (**h**) cleaved ATF6 (50 kDa) compared to its total protein or GAPDH in RAECs. Data are expressed as the mean ± SEM, n = 4. # *P <* 0.05 vs. NG. * *P* < 0.05 vs. HG.

To confirm TMS is acting on endothelial cells, protein expression was determined in RAECs. After treatment with high glucose (44 mM) for 48 h, the phosphorylation of AMPKα at Thr172, the phosphorylation of eNOS at Ser1177, and expression of SIRT1 was all suppressed while significantly reversed by co-treatment with TMS (10 and 50 μM) (Figure 5a–c). Additionally, high glucose stimulation decreased the nitrite production but treatment with TMS remarkably, alleviated it (Figure 5d), indicating an improved NO bioavailability in RAECs. TMS also down-regulated ER stress markers such as GRP78, phosphorylation of eIF2α at Ser52, spliced XBP1, and cleaved ATF6 in RAECs upon high glucose exposure (Figure 5e–h).

### 3.5. TMS Treatment Alleviates Endothelial Dysfunction and Oxidative Stress in Diet-Induced Obese (DIO) Mice

**Figure 6:**
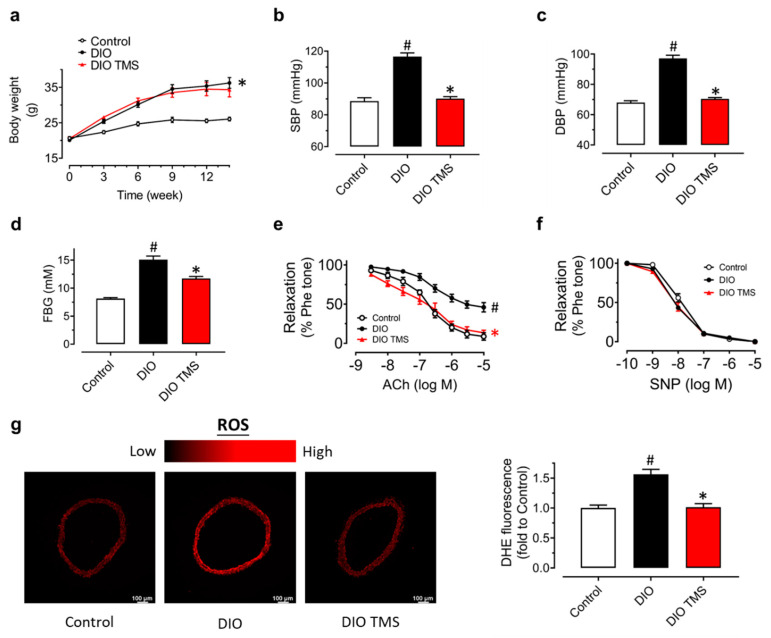
Effects of TMS in diet-induced obese (DIO) mice. (**a**) Changes in body weight in mice fed with high-fat or normal chow diet for 14 weeks and administered with vehicle (HS-15) and TMS at 10 mg/kg body weight daily via oral gavage in the last 4 weeks. (**b**,**c**) Changes in systolic (SBP) and diastolic (DBP) blood pressures tested by the tail-cuff method. (**d**) Comparison of fasting blood glucose (FBG) measured after fasting the mice for 6 h. (**e**) Vaso-protective effect of TMS treatment on ACh-induced EDRs in aortas from DIO mice. (**f**) SNP-induced endothelium-independent relaxation in mouse aortas. (**g**) Representative images and summarized graph showing DHE intensity (oxidative stress indicator) in mouse aortas from DIO mice. Data are expressed as the mean ± SEM, n = 4–5. # *P* < 0.05 vs. Control. * *P* < 0.05 vs. DIO.

Because TMS exhibits positive effect against high glucose-induced endothelial dysfunction ex vivo, animal experiments were designed to explore its effect on endothelial dysfunction in vivo. Diet-induced obese (DIO) mice were orally administered with vehicle (HS-15) and TMS (10 mg/kg/day) for 4 weeks. Compared to the control mice fed with normal chow, DIO mice that were fed with high-fat diet for 14 weeks had much higher body weight but TMS treatment showed no effect on the body weight (Figure 6a). Systolic (SBP) and diastolic (DBP) blood pressures were notably upregulated in DIO mice while TMS treatment rescued them significantly (Figure 6b,c). From the result of fasting blood glucose (FBG) of the three groups, the mean glucose level of DIO mice was higher than 11 mM which suggested a successful establishment of a diabetic model (Figure 6d). TMS treatment significantly ameliorated the FBG even though the effect was not strong enough to return the level to be comparable with the control group.

Moreover, ACh-induced EDRs were impaired in DIO mice but greatly improved by chronic TMS treatment (Figure 6e). SNP-induced endothelium-independent relaxations in mouse aortas remained unaffected (Figure 6f). To further examine the anti-oxidative effect of TMS that had been tested in RAECs, DHE staining of the aortas from DIO mice was applied to measure the ROS production (Figure 6g). Feeding with a high-fat diet resulted in elevated ROS level in aortas while chronic TMS treatment attenuated the oxidative stress. Taken together, chronic TMS treatment ameliorated endothelial dysfunction and oxidative stress in aortas from DIO mice.

### 3.6. TMS Treatment Enhances the AMPK/SIRT1/Enos Pathway and Attenuates ER Stress in Aortas from DIO Mice

**Figure 7:**
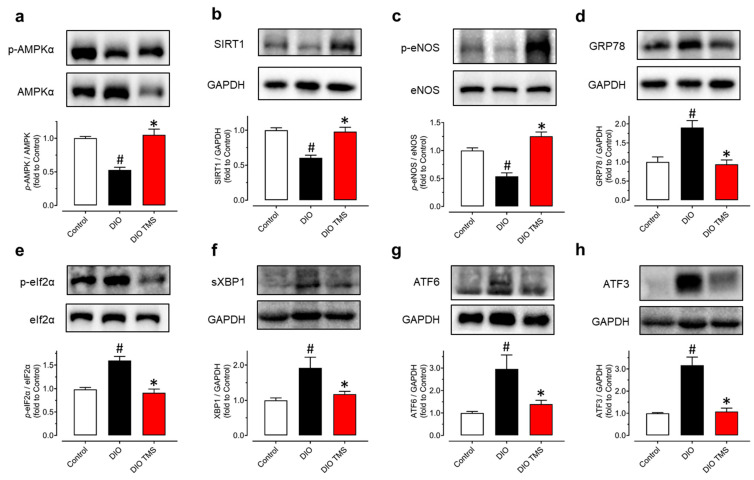
Chronic TMS treatment stimulates the AMPK/SIRT1/eNOS pathway and reduces ER stress in DIO mice. Western blot results showing (**a**) phosphorylation of AMPKα at Thr172 (p-AMPKα; 62kDa), (**b**) expression of SIRT1 (120 kDa), (**c**) phosphorylation of eNOS at Ser1177 (p-eNOS; 140 kDa), (**d**) GRP78 (78 kDa), (**e**) the phosphorylation of eIF2α at Ser52 (p-eIF2α; 38 kDa), (**f**) spliced XBP1 (sXBP1; 56 kDa), (**g**) cleaved ATF6 (50 kDa) and (**h**) ATF3 (21 kDa) as compared to their corresponding total proteins or GAPDH (37 kDa) in mouse aortas from three groups of mice. Data are expressed as the mean ± SEM, n = 4–5. # *P* < 0.05 vs. Control. * *P* < 0.05 vs. DIO.

It had been confirmed that TMS upregulated the AMPK/SIRT1/eNOS pathway and decreased ER stress both in mouse aortas and RAECs stimulated in a hyperglycemic condition. In aortas from DIO mice, the phosphorylation of AMPKα at Thr172, the phosphorylation of eNOS at Ser1177, and expression of SIRT1 were decreased (Figure 7a–c) while ER stress markers including GRP78, phosphorylation of eIF2α at Ser52, spliced XBP1, cleaved ATF6 and ATF3 were increased when compared to control mice (Figure 7d–h). Consistent with the in vitro results in mouse aortas and RAECs, TMS treatment reversed all these changes in DIO mice. These findings validated the effect of TMS on enhancing the AMPK/SIRT1/eNOS pathway and reduction in ER stress in vivo.

## 4. Discussion

In this study, we employed multiple methods to determine the protective effect of TMS against endothelial dysfunction in diabetic and obese mice. All the results suggested that treatment with TMS restored the impaired endothelial function both in a hyperglycemic condition ex vivo and in DIO mice via activation of the AMPK/SIRT1/eNOS signaling pathway and reduction in ER stress and oxidative stress.

Vascular endothelium plays an essential role in maintaining vascular homeostasis which can be disrupted by many risk factors such as hyperglycemia, mediating the development and progression of cardiovascular diseases and the vascular complications linked with metabolic syndromes [23]. NO was recognized as one of most important relaxing mediators derived from endothelium. Vascular endothelium-derived NO accounts not only for the relaxation of vasculature, but also for platelet adhesion and the inhibition of platelet aggregation [24,25,26]. Endothelial dysfunction is a structural and functional impairment of vascular endothelium. Extensive evidence has supported the idea that decreased eNOS activity but increased ER stress and oxidative stress lead to reduction in NO bioavailability and endothelial dysfunction. Our present study firstly treated mouse aortas with high glucose to mimic the hyperglycemic condition in diabetes and found that TMS significantly alleviated the high glucose-triggered impairment in endothelium-dependent vasodilation. Different concentrations of high glucose were applied depending on the species as described in previous studies, 30 mM for mouse aortas and 44 mM for RAECs [18,27].

Many modulators regulate NO bioavailability and endothelial function. AMPK is reported to regulate many signaling cascades and is involved in SIRT1 activation. Phosphorylation of eNOS at serine 1177 is associated with enhanced eNOS activity and AMPK can also phosphorylate eNOS at serine 1177 [28]. SIRT1, a homologue of silence information regulator (Sir2) protein, has been proven to promote EDRs through deacetylating eNOS and stimulating eNOS activity [29]. In ECs, SIRT1 modulates endothelial homeostasis and normal vascular functions via regulating not only eNOS activity, but also Ang II type 1 receptor (AT1R), p53, and forkhead box O (FOXO) 1 [30]. Additionally, it is reported that oxidative stress can downregulate SIRT1, contributing to acetylation of eNOS and reduction in NO production in ECs [31]. Previous studies have proved that resveratrol exhibits vaso-protective effects by activation of AMPK and SIRT1 [20,32,33], so we used AMPK inhibitor Compound C and SIRT1 inhibitor EX527 to examine whether TMS, having a similar structure, might have similar activities to RSV. The results showed that the vaso-protective effect of TMS was abolished by these two inhibitors, indicating that both AMPK and SIRT1 take part in the underlying mechanisms of TMS. Likewise, the results from Western blotting suggested that TMS ameliorated high glucose-induced endothelial dysfunction via stimulating the AMPK/SIRT1/eNOS pathway and reducing ER stress. We also compared the effects of TMS and RSV on EDRs. The results show that both compounds improved EDRs in a dose-dependent manner. Of note, TMS treatment improved vasodilation at a concentration of 1 μM while the effective concentration of RSV was 20 μM. This suggests that TMS might exert a more potent vaso-protective effect than RSV.

ER stress has been proven by many studies to be associated with many human pathologies including atherosclerosis, obesity, diabetes, inflammation, neurodegenerative disorders, and cancer [34]. It can be triggered by high glucose, oxidative stress, ischemia, Ca^2+^ overload, and some other risk factors. Activation of AMPK is an important defensive response to inhibit ER stress [35]. Excessive production of ROS leads to oxidative stress which is considered a pivotal pathophysiology for cardiovascular diseases including atherosclerosis and endothelial dysfunction. Oxidative stress contributes to the endothelial dysfunction via several molecular mechanisms, including uncoupling of eNOS, upregulation of endothelin-1 (a powerful endogenous vasoconstrictor) with subsequent production of superoxide/hydrogen peroxide, and activation of NADPH oxidase by angiotensin II [36]. Hyperglycemic conditions increase the formation of electron-transferring compounds like NADPH, and this will result in increased formation of ROS. In addition, increased NADPH oxidase-derived ROS contributes to the oxidative depletion of tetrahydrobioterin (BH_4_) which is essential for eNOS uncoupling [37,38]. Emerging evidence proves that both AMPK and SIRT1 are involved in the suppression of ROS production to protect endothelial function in cardiovascular and metabolic diseases [39].

Previous studies have illustrated some mechanisms mediated by TMS in various cell types and disease conditions [9,11,13,40,41,42]. Notably, SIRT1 plays an important role in its protective effects against hyperglycemia or obesity. Our findings are consistent with these observations. We found that the activation of AMPK and SIRT1 was involved in the vaso-protective effect of TMS against high-glucose-induced endothelial dysfunction. Exposure to high glucose leads to impaired EDRs, down-regulation of phosphorylation of AMPK at Ser172 and eNOS at Ser1177 and SIRT1 expression, decreased NO bioavailability, and up-regulated ER stress in mouse aortas ex vivo, while co-incubation with TMS reversed these impairments. Similar results were obtained in primary culture of RAECs, indicating that TMS acted on endothelial cells. Additionally, to compare the anti-oxidative effect between TMS and RSV, DHE staining was performed in RAECs. The results revealed that TMS had similar anti-oxidative effect with RSV since they both exhibited inhibitory effects against ROS production at 10–50 μM but not 1 μM. The reduction in ROS by TMS might be attributed to the activation of the AMPK/SIRT1/eNOS pathway. However, we cannot rule out the involvement of other signaling pathways or enzymes. The detailed mechanisms modulating its effect against oxidative stress is yet to be investigated in future studies.

Based on the results ex vivo and in vitro, we further explored the beneficial effects of TMS in diabetic and obese mice. Chronic treatment of TMS (10 mg/kg/day, 4 weeks) by oral gavage showed no effect on the body weight but attenuated hyperglycemia to some extent. Its function on insulin sensitivity and glucose homeostasis is not fully understood, but can be explored in the future. The in vivo study suggested significant benefits of TMS in vascular function, normalizing blood pressure and improving EDRs in aortas from DIO mice. Moreover, ROS levels in aortas from DIO mice were also reduced which is in line with the anti-oxidative effect in vitro. Western blotting results showed that chronic TMS treatment increased phosphorylation of AMPK at Ser172 and eNOS at Ser1177 and SIRT1 expression while downregulating ER stress markers in DIO mice. Taken together, TMS exerts vaso-protective effects through activation of the AMPK/SIRT1/eNOS pathway and suppression of ER stress and oxidative stress. Previous studies have demonstrated that RSV exerted benefiical effects, such as protecting endothelial function when treated at 20 mg/kg/day or higher dosages [8,20]. Currently, we found that TMS could ameliorate the diabetes-associated endothelial dysfunction at a lower dosage 10 mg/kg/day. Previous pharmacokinetic studies suggested that TMS exhibited more favarable results than RSV [43,44]. RSV possessed a very short-half life and limited plasma exposure due to extensive phase II metabolism such as sulfation and glucuronidation. Notably, its clearance was about eight to nine fold faster than that of TMS. Stability of TMS was improved, owing to the complete methoxylation of the hydroxyl groups when compared with RSV. Consequently, the plasma exposure of TMS after oral administration would be much more abundant than that of RSV. Future extensive efforts are still needed to compare the effectiveness of TMS and RSV at different dosages in vivo.

## 5. Conclusions

TMS exerted protective effects against endothelial dysfunction (consisting of ER stress and oxidative stress induced by high glucose), in both mouse aortas ex vivo and RAECs in vitro, through activation of the AMPK/SIRT1/eNOS pathway, which was further confirmed in vivo in a high-fat, diet-induced, diabetic and obese mouse model. The present study provides novel evidence supporting the beneficial effects of TMS in protecting against endothelial dysfunction associated with metabolic disorders.

## Data Availability

The data presented in this study are available on request from the corresponding author.
